# PSYSCAN multi-centre study: baseline characteristics and clinical outcomes of the clinical high risk for psychosis sample

**DOI:** 10.1038/s41537-025-00598-x

**Published:** 2025-04-17

**Authors:** Stefania Tognin, Sandra Vieira, Dominic Oliver, Alexis E. Cullen, Mathew J. Kempton, Paolo Fusar-Poli, Andrea Mechelli, Paola Dazzan, Kate Merritt, Arija Maat, Lieuwe de Haan, Stephen M. Lawrie, Thérèse van Amelsvoort, Celso Arango, Barnaby Nelson, Silvana Galderisi, Rodrigo Bressan, Jun Soo Kwon, Romina Mizrahi, the PSYSCAN Consortium, Andrea Mechelli, Arija Maat, Barnaby Nelson, Rodrigo Bressan, Jun Soo Kwon, Paolo Fusar-Poli, Matthew Kempton, Gemma Modinos, Helen Baldwin, Kate Merritt, Fiona Coutts, Emily Hird, Paola Dazzan, George Gifford, Natalia Petros, Mathilde Antoniades, Andrea De Micheli, Sandra Vieira, Tom Spencer, Rene Kahn, Erika van Hell, Inge Winter, Lieuwe de Haan, Frederike Schirmbeck, Benedicto Crespo-Facorro, Diana Tordesillas-Gutierrez, Esther Setien-Suero, Rosa Ayesa-Arriola, Paula Suarez-Pinilla, Victor Ortiz Garcia-de la foz, Birte Glenthøj, Mikkel Erlang Sørensen, Bjørn H. Ebdrup, Karen Tangmose, Helle Schæbel, Egill Rostrup, Stephen Lawrie, Colm McDonald, Brian Hallahan, Dara Cannon, James McLoughlin, Martha Finnegan, Oliver Gruber, Anja Richter, Bernd Krämer, Therese van Amelsvoort, Bea Campforts, Machteld Marcelis, Claudia Vingerhoets, Celso Arango, Covandonga M. Díaz-Caneja, Miriam Ayora, Joost Janssen, Roberto Rodríguez-Jiménez, Marina Díaz-Marsá, Tilo Kircher, Irina Falkenberg, Florian Bitsch, Jens Sommer, Patrick McGorry, Paul Amminger, Meredith McHugh, Silvana Galderisi, Armida Mucci, Paola Bucci, Giuseppe Piegari, Daria Pietrafesa, Sara Patriarca, André Zugman, Ary Gadelha, Graccielle Rodrigues da Cunha, Kang Ik Kevin Cho, Tae Young Lee, Minah Kim, Sun-Young Moon, Silvia Kyungjin Lho, Mark Weiser, Romina Mizrahi, Michael Kiang, Cory Gerritsen, Margaret Maheandiran, Sarah Ahmed, Ivana Prce, Jenny Lepock, Gabriele Sachs, Matthäus Willeit, Marzena Lenczowski, Ullrich Sauerzopf, Ana Weidenauer, Julia Furtner-Srajer, Matthias Kirschner, Anke Maatz, Achim Burrer, Philipp Stämpfli, Naemi Huber, Wolfram Kawohl, Rene S. Kahn, Philip McGuire

**Affiliations:** 1 Department of Psychosis Studies, Institute of Psychiatry, Psychology & Neuroscience, King’s College London, De Crespigny Park, Denmark 458 Hill, SE5 8AF London, UK; 2 Outreach and Support in South London (OASIS), South London and Maudsley NHS Foundation Trust, London, UK; 3 Department of Radiology, Lausanne University Hospital and University of Lausanne (CHUV-UNIL), Lausanne, Switzerland; 4 Center for Research in Neuropsychology and Cognitive Behavioural Intervention, Faculty of Psychology and Educational Sciences, University of Coimbra, Coimbra, Portugal; 5 Department of Psychiatry, University of Oxford, Warneford Hospital, Oxford, OX3 7JX UK; 6 NIHR Oxford Health Biomedical Research Centre, Oxford, OX3 7JX UK; 7 OPEN Early Detection Service, Oxford Health NHS Foundation Trust, Oxford, UK; 8 Department of Brain and Behavioral Sciences, University of Pavia, Pavia, Italy; 9 National Institute for Health Research, Maudsley Biomedical Research Centre, South London and Maudsley NHS Foundation Trust, London, UK; 10 Department of Psychiatry and Psychotherapy, Ludwig-Maximilian University, Munich, Germany; 11 Department of Psychological Medicine, Institute of Psychiatry, Psychology and Neuroscience, King’s College London, London, UK; 12 Division of Psychiatry, University College London, London, UK; 13 University Medical Center, Division of Neurosciences, Department of Psychiatry, Heidelberglaan 100, 3584 CX Utrecht, The Netherlands; 14 Amsterdam UMC, University of Amsterdam, Psychiatry, Department Early Psychosis, Meibergdreef 9, Amsterdam, The Netherlands; 15 Division of Psychiatry, University of Edinburgh, Royal Edinburgh Hospital, Edinburgh, EH10 5HF UK; 16 Department of Psychiatry and Neuropsychology, Maastricht University, Maastricht, The Netherlands; 17 Mondriaan Mental Health Care, Heerlen, The Netherlands; 18 Departmento de Psiquiatria, Instituto de Investigacion Sanitaria Hospital 12 de Octubre (imas12), Madrid, Spain; 19 CIBERSAM (Biomedical Research Networking Centre in Mental Health), Madrid, Spain; 20 Orygen, 35 Poplar Road, Parkville, VIC Australia; 21 Centre for Youth Mental Health, The University of Melbourne, Parkville, VIC Australia; 22 Department of Mental and Physical Health and Preventive Medicine, University of Campania Luigi Vanvitelli, Largo Madonna delle Grazie, 80138 Naples, Italy; 23 Department of Psychiatry, Interdisciplinary Lab for Clinical Neurosciences (LiNC), Universidade Federal de Sao Paulo (UNIFESP), Sao Paulo, Brazil; 24 Department of Psychiatry, Seoul National University College of Medicine, 101 Dahakno, Jongno-gu, Seoul, Korea; 25 Department of Psychiatry, McGill University, Montreal, Canada; 26 Department of Psychiatry and Behavioral Health System, Icahn School of Medicine at Mount Sinai, One Gustave L. Levy Place, Box 1230, New York, NY 10029-6574 USA; 27 Department of Psychiatry, Marqués de Valdecilla University Hospital, IDIVAL. School of Medicine, University of Cantabria, Santander, Spain; 28 CIBERSAM, Centro Investigación Biomédica en Red Salud Mental, Madrid, Spain; 29 Centre for Neuropsychiatric Schizophrenia Research (CNSR) & Centre for Clinical Intervention and Neuropsychiatric Schizophrenia Research (CINS), Mental Health Centre Glostrup, University of Copenhagen, Glostrup, Denmark; 30 University of Copenhagen, Faculty of Health and Medical Sciences, Dept. of Clinical Medicine, Copenhagen, Denmark; 31 Functional Imaging Unit (FIUNIT), Rigshospitalet Glostrup, University of Copenhagen, Glostrup, Denmark; 32 Centre for Neuroimaging & Cognitive Genomics (NICOG), NCBES Galway Neuroscience Centre, National University of Ireland Galway, H91 TK33 Galway, Ireland; 33 Section for Experimental Psychopathology and Neuroimaging, Department of General Psychiatry, Heidelberg University, Heidelberg, Germany; 34 GGZE Mental Health Care, Eindhoven, The Netherlands; 35 Servicio de Psiquiatría del Niño y del Adolescente, Hospital General Universitario Gregorio Marañon, Universidad Complutense Madrid, Madrid, Spain; 36 Centro de Investigación Biomédica en Red de Salud Mental, Madrid, Spain; 37 Hospital Clínico de San Carlos, Universidad Complutense, Centro de Investigación Biomédica en Red de Salud Mental (CIBERSAM), Madrid, España; 38 Department of Psychiatry, University of Marburg, Rudolf-Bultmann-Straße 8, D-35039 Marburg, Germany; 39 Department of Psychiatry, University of Campania Luigi Vanvitelli, Largo Madonna delle Grazie, 80138 Naples, Italy; 40 Department of Psychiatry, Sheba Medical Center, Tel Hashomer, 52621 Israel; 41 Sackler School of Medicine, Tel Aviv University, Tel Aviv, Israel; 42 Institute of Medical Science, University of Toronto, 1 King’s College Circle Room 2374, Toronto, ON M5S 1A8 Canada; 43 Centre for Addiction and Mental Health, 250 College Street, Toronto, ON M5T 1R8 Canada; 44 Department of Psychology, University of Toronto, 100 St. George Street 4th Floor, Toronto, ON M5S 3G3 Canada; 45 Department of Psychiatry and Psychotherapy, 1090 Vienna, Austria; 46 Medical University of Vienna, Department of Biomedical Imaging and Image-guided Therapy Währingergürtel 18-20, 1090 Vienna, Austria; 47 Department of Psychiatry, Psychotherapy and Psychosomatics, Psychiatric Hospital, University of Zurich, Zurich, Switzerland; 48 Department for Psychiatry and Psychotherapy, Psychiatric Services Aargau, Brugg, Switzerland

**Keywords:** Psychosis, Biomarkers

## Abstract

Predicting outcomes in individuals at clinical high risk (CHR) of developing psychosis remains challenging using clinical metrics alone. The PSYSCAN project aimed to enhance predictive value by integrating data across clinical, environmental, neuroimaging, cognitive, and peripheral blood biomarkers. PSYSCAN employed a naturalistic, prospective design across 12 sites (Europe, Australia, Asia, Americas). Assessments were conducted at baseline, 3, 6, and 12 months, with follow-ups at 18 and 24 months to evaluate clinical and functional outcomes. The study included 238 CHR individuals and 134 healthy controls (HC). At baseline, CHR and HC groups differed significantly in age, education, IQ, and vocational and relationship status. Cannabis and tobacco use did not significantly differ between groups, however CHR individuals had higher proportion of moderate to high risk of tobacco abuse. A substantial portion of the CHR sample met DSM criteria for anxiety (53.4%) and/or mood disorders (52.9%), with some prescribed antidepressants (38.7%), antipsychotics (13.9%), or benzodiazepines (16.4%). Over the follow-up period, 25 CHR individuals (10.5%) transitioned to psychosis. However, the CHR group as a whole showed improvements in functioning and attenuated psychotic symptoms. Similar to other recent multi-centre studies, the CHR cohort exhibits high comorbidity rates and relatively low psychosis transition rates. These findings highlight the clinical heterogeneity within CHR populations and suggest that outcomes extend beyond psychosis onset, reinforcing the need for broader prognostic models that consider functional and transdiagnostic outcomes.

## Introduction

Psychotic disorders usually emerge in late adolescence and early adulthood and can be personally and socially devastating due to the potential for life-long disability^1–3^. This has led to a worldwide effort to develop strategies for early identification, intervention, and prevention of psychosis^4–8^. A critical step towards this goal has been the operationalization of a “clinical high risk” (CHR) state^9^. People with this clinical syndrome typically present with attenuated symptoms, or less commonly, a brief psychotic episode that spontaneously resolves and/or genetic vulnerability in the context of a recent decline in functioning or chronic low functioning^10^. In addition to psychosis-related features, CHR individuals often exhibit a heterogeneous and complex clinical profile. Up to three-quarters have at least one comorbid mental health disorder, most commonly anxiety and mood disorders, but also including trauma-related and personality disorders^11^. Substance use is also prevalent in this group, with tobacco and cannabis being the most frequently reported substances^12–14^. The risk of developing psychosis among people presenting with a CHR state is high, and can vary from 12 to 43%, according to the nature of the sample, how it was ascertained, and the length of follow up^15^. However, most transitions occur during the first two years^16^. There is also a growing interest towards other outcomes, such as symptom severity^17–19^ and socio-occupational functioning impairments^20^. Although evidence is mixed, improvements in these areas are often seen within the first two years but are typically not sustained over the longer term^21^. Among the majority who do not develop psychosis, many already meet criteria for another mental health disorder or will subsequently experience other mental health issues or mental-health-related disability^22,23^.

Despite the heterogeneity of clinical presentation and outcomes, meeting CHR status remains the most reliable risk factor for psychosis^24^. This underscores the importance of studying the CHR state not only as a predictor of psychosis but also as a critical period for understanding the early alterations associated with the disorder. However, implementing such studies faces significant challenges. The low prevalence of CHR and transition rates in the general population compared to specialised CHR services^25,26^, coupled with the growing yet still limited availability of such services globally^27^, pose significant obstacles to recruitment. Furthermore, the large sample needed to disentangle specific pathways to psychosis onset in a heterogeneous group^28^ is difficult to achieve in single-centre studies within a reasonable timeframe. The inherent longitudinal nature of CHR studies adds further complexity. In response to these challenges, large-scale, multicentre, longitudinal studies such as NAPLS^5^, PRONIA^4^, and the ongoing AMP SZ^29^ have been developed. These collaborative efforts are designed to overcome the limitations of single-centre studies by pooling resources, harmonising methodologies, and increasing sample sizes to advance the understanding of CHR populations and improve early intervention strategies.

The PSYSCAN project is an international, naturalistic, prospective study focused on individuals at the early stages of psychosis. It encompasses a broad range of neuroimaging, clinical, cognitive, biological, and genetic variables collected at baseline and at follow-up^30^. CHR patients have been recruited from diverse regions, namely Europe, North America, South America, Asia, and Australia. This global reach captures variations in healthcare systems, cultural contexts and patterns of help-seeking, essential for advancing early detection and prevention of psychosis. This complements other large studies such as NAPLS that was carried out in one country^5^. For example, the NAPLS risk calculator to transition to psychosis^6^ generalised well to other North American sites^31^ but had a modest performance in a sample from Shanghai^32^. In addition to a wide geographical coverage, the PSYSCAN’s protocol includes a novel brief computerised cognitive battery for psychosis^33^, advanced neuroimaging techniques such as diffusion tensor imaging, and omics data to investigate less explored biomarkers in CHR such as keratinocytes, lipidomics, and redox. A further issue of past large studies in the early psychosis population is low retention rates. To overcome this issue and improve the quality and quantity of follow up data, the PSYSCAN protocol included close assessment time-points. The PSYSCAN study comprises three cohorts: individuals with a recent first episode of psychosis^34^, CHR and healthy controls (HC). The present paper describes how the CHR and healthy control samples were recruited and assessed, their sociodemographic and clinical characteristics at baseline, and the clinical and functional outcomes at follow up.

## Material and methods

### Study design and samples

A multi-centre, naturalistic, longitudinal design was employed. CHR individuals were assessed at baseline, 3, 6, 12, 18, and 24 months (Supplementary Table S1). The PSYSCAN project also involved the recruitment of a first episode psychosis patients (FEP) cohort, which is described in a separate study^34^, with healthy controls (HC) serving as a shared control group for both the CHR and FEP cohorts. HCs were assessed at baseline, 6, and 12 months (Supplementary Table S2).

CHR individuals were recruited from July 2016 to December 2019 at 10 sites: London, Amsterdam, Maastricht, Madrid, Naples, Melbourne, Seoul, Hong Kong, Toronto, Sao Paulo. The recruiting sites were all experienced in providing health care for CHR individuals. All participants were help-seeking and were under the care of a clinical service at the time of inclusion. A subset of 8 sites involved in the larger study (i.e. including FEP recruiting sites), additionally recruited HC individuals: London, Edinburgh, Utrecht, Maastricht, Amsterdam, Madrid, Seoul, and Melbourne. HC were recruited through advertisement and attempts were made to include HC who were similar in terms of sociodemographic characteristics (age, sex, and ethnicity) to the CHR and FEP cohorts.

Inclusion criteria for the CHR and HC cohorts were: age 16–40 years (except for one site, Madrid, who recruited 14–40), and ability to provide written informed consent, assent and written informed consent of parents and/or legal guardians if 14–17 years old (depending on local laws and regulations). The age lower limit was set to 16 as it aligns with research consent practices across sites. The broader upper age limit accommodates the inclusion of healthy controls shared with the FEP cohort, while the lower minimum age in Madrid results from the local collaboration between paediatric and adult psychiatry services. Additionally, CHR participants were required to meet criteria for either the Comprehensive Assessment of At-Risk Mental State (CAARMS) criteria^9^ or *basic symptoms* assessed using the Schizophrenia Proneness Instrument (SPI-A)^35^ (see Supplementary Materials for details). Extensive measures were undertaken to ensure consistency in clinical evaluations across sites using either CAARMS or SPI-A including training and monthly monitoring calls to discuss cases with experienced raters and clinicians^30^.

Exclusion criteria for CHR and HC included any previous neurosurgery or neurological disorder, including epilepsy; history of head injury resulting in unconsciousness lasting at least 1 hour; pregnancy, any other contraindications for MRI; refusing to have blood drawn and/or MRI performed; inability to fully comprehend the purpose of the study or make a rational decision whether or not to participate; having an estimated IQ < 70; having received antipsychotic medication for > 30 days (cumulative number of days) in the 3 months prior to baseline assessments (including self-ratings and screening assessments), at doses that would be adequate for treating a first episode of psychosis (i.e. excludes very low doses); a past episode of frank psychosis lasting > 7 days. In addition, HCs were excluded if they reported a lifetime history of any DSM-IV Axis-I or Axis-II (borderline, paranoid and schizotypal) disorder; met CAARMS^9^ or SPI-A^35^ criteria; had a first-degree relative with a lifetime history of affective or non-affective psychosis (defined by treatment or diagnosis); or reported previous use of antipsychotic medication or current use of any psychoactive medication. Ethical approval was obtained from each site’s local research ethics committee. The study was conducted in accordance with the Declaration of Helsinki. All participants provided written informed consent.

### Assessments and measures

The comprehensive schedule of assessments, which were translated in each participating site’s main language, is listed in supplementary Table S1 (CHR) and **S2** (HC). Both CHR individuals who did and did not develop psychosis followed the same assessment schedule. Those individuals who developed psychosis during the follow-up period were approached to complete all the planned visits listed in supplementary Table S1 and were not included in the FEP sample. Due to the start of the COVID-19 pandemic, assessments in 2020-21 were mostly completed online using telephone, or other remote platforms. To maximize retention and data completeness, follow-up assessments were extended beyond 24 months when feasible and/or necessary. This occurred when (1) COVID-19 delays disrupted scheduled assessments, (2) participants missed their 24-month follow-up but agreed to later contact, or (3) they remained engaged with clinical services beyond 24 months. In these cases, data were collected via face-to-face assessments or electronic health records.

Sociodemographic data were collected at baseline. Medical and psychiatric history were also evaluated at baseline and updated during subsequent assessments using a semi-structured interview. Psychopathology in CHR individuals was assessed at baseline, 6, 12, and 24 months using a two-part tool (Clinical High Risk Assessment Tool; CHRA, Part 1 and Part 2). Additionally, interim assessments at 3 and 18 months were conducted to determine if the individual had transitioned to psychosis. CHR state, symptoms remission (defined as no longer meeting criteria for CHR state) and transition to psychosis during the study were assessed using the Psychotic Symptoms module of the CAARMS^9^ and SPI-A)^35^. Functioning was assessed using Social and Occupational Functioning Scale (SOFAS)^36^. Criteria for other mental health disorders were assessed using the Structured Clinical Interview for DSM-IV Disorders -I (SCID-I^37^) and SCID-II (paranoid, schizotypal, and borderline personality disorder modules)^38^). Inter-rater reliability and consistency across sites were ensured throughout via monthly or bimonthly group monitoring calls where new cases were discussed and CAARMS^9^ and SPI-A^35^ scoring confirmed by experienced clinicians and trainers.

Substance use was assessed with the Alcohol, Smoking and Substance Involvement Screening Test 3.0 (ASSIST^39^). Cannabis use, age of onset, frequency, quantity, duration and substance preference was assessed using the adapted Cannabis Experience Questionnaire^40^. IQ was assessed using a short version of the WAIS^41^.

Transition to psychosis was defined either psychometrically using CAARMS criteria or clinically using Diagnostic and Statistical Manual of Mental Disorders (DSM)- or the International Classification of Diseases (ICD)-defined diagnoses or accepted referrals to early intervention services recorded in electronic health records (EHRs).

### Statistical analysis

All data collected were transferred to a central database managed by IXICO (https://ixico.com) for processing and analysis throughout the project. The present study focuses on the analyses on key baseline socio-demographic in the CHR and HC samples and clinical data in the CHR sample, as well as follow up data on CHR functioning and symptom severity. Data analysis was performed using python 3.5 and R 4.2.2. The threshold for statistical significance was *p* < 0.05. Independent samples t-tests and Chi-square tests were used to compare CHR and HC on continuous and categorical variables, respectively. Symptom severity was indexed by multiplying severity and frequency scores for each of the four CAARMS positive items. The total CAARMS positive score was the sum of these individual CAARMS positive items. Functional remission was defined as a SOFAS score >68 as defined in previous studies^42^. The cumulative incidence of psychosis was visualized with the Kaplan–Meier failure function (1—survival)^43^ and Greenwood 95% confidence intervals (CIs)^44^, conducted using the “survival” (version 3.5-7) and “survminer” (version 0.4.9) packages. The difference in incidence between individuals followed up using psychometric assessment (CAARMS) and EHRs was compared using a Cox proportional hazards model, following confirmation of the proportional hazards assumption being met using the Global Schoenfeld Test^45^. Cox proportional hazards models were run both unadjusted and adjusted for sociodemographic (age, sex and ethnicity) or clinical variables (baseline CAARMS positive scores and baseline SOFAS scores) with significant group differences (assessed with independent t-test for continuous variables and Fisher’s exact test for categorical variables). Differences between baseline and follow-up positive CAARMS and SOFAS scores were assessed using paired two-tailed t-tests. To mitigate against potential survivorship bias, we presented the baseline descriptives for all participants and then compared the difference between scores from the baseline assessment and each participant’s final follow-up assessment, only in participants who attended at least one follow-up assessment.

## Results

### Recruitment and retention

**Fig. 1:**
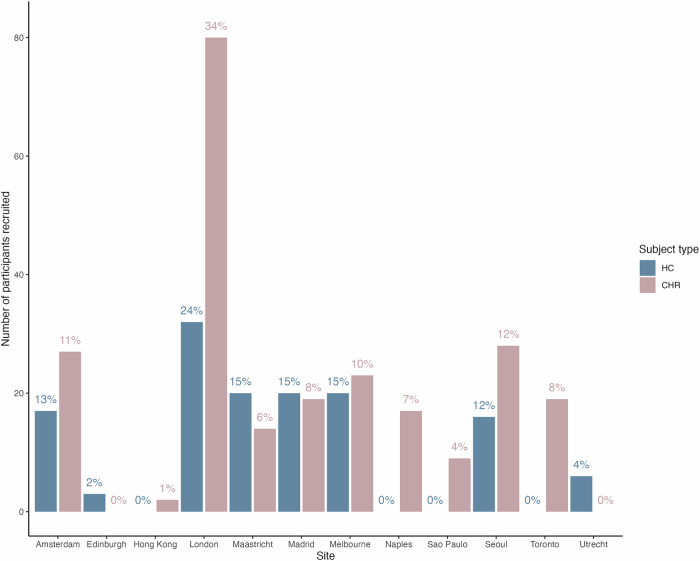
Number of HC and CHR recruited per site. Percentage of the total HC and CHR samples recruited at each site are presented above each bar.

From July 2016 to December 2019, 372 participants were recruited across the 12 sites. This included 238 CHR and 134 HC (Table 1). CHR participants were recruited from 10 different sites, with a mean of 23.8 subjects enrolled per site (Fig. 1). In general, recruitment was highest at sites where there was a well-established clinical early detection service. The HC sample was recruited from 8 sites, with a mean of 16.8 subjects enrolled per site.

**Table 1:** Sociodemographic characteristics of individuals at clinical high-risk and healthy controls.

					HC (*N* = 134)	CHR (*N* = 238)	*p*
Age, mean (SD)					23.7 (4.4)	22.4 (4.7)	**0.005**
Sex, *n* (%)		Female			54 (40.3)	110 (46.4)	0.303
		Male			80 (59.7)	127 (53.6)	
		Missing			0	0	
Ethnicity, *n* (%)		Asian Indian			8 (6.0)	8 (3.8)	0.163
		Black			11 (8.2)	24 (10.1)	
		East Asian			31 (23.1)	39 (16.5)	
		Other			7 (5.2)	24 (10.1)	
		White			77 (57.4)	142 (59.9)	
		Missing			0 (0)	1 (0.4)	
Relationship status, *n* (%)		In a relationship			55 (43.0)	54 (23.7)	**0.001**
		Other			2 (1.6)	2 (0.9)	
		Single/divorced/separated			71 (55.5)	172 (75.4)	
		Missing			6 (4.5)	10 (4.2)	
Current living conditions, *n* (%)		Living alone			18 (13.7)	25 (10.7)	0.167
		Living with family/partner/friends/other			112 (85.5)	199 (85.4)	
		Other			1 (0.8)	9 (3.9)	
		Missing			3 (2.2)	5 (2.1)	
Education (years), mean (SD)					15.9 (3.1)	14.1 (3.3)	**<0.001**
		Missing			0	0	
Student, *n* (%)		No			46 (35.9)	120 (51.3)	**0.011**
		Yes, full time			71 (55.5)	92 (39.3)	
		Yes, part time			11 (8.6)	22 (9.4)	
		Missing			6 (4.5)	4 (1.7)	
Current employment, *n* (%)		Employed full-time			29 (26.6)	32 (15.9)	**<0.001**
		Employed part-time			55 (50.5)	68 (33.8)	
		Unemployed			25 (22.9)	101 (50.2)	
		Missing			25 (18.7)	37 (15.5)	
Father - years of education, mean (SD)					14.8 (4.1)	14.3 (4.0)	0.301
		Missing			79		
Mother - years of education, mean (SD)					15.0 (3.8)	14.2 (4.2)	0.104
		Missing			68		
IQ, mean (SD)					112.6 (15.7)	105.0 (17.8)	**<0.001**
		Missing			38		

*CHR* clinical high risk for psychosis; *HC* healthy controls; *IQ* intelligence quotient; *SD* standard deviation. Independent samples *t*-tests and Chi-square tests were used to compare CHR and HC on continuous and categorical variables, respectively.

Statistically significant comparisons are shown in bold.

77.9% of the CHR sample were followed-up at 3 months, 75.0% at 6 months, 61.4% at 12 months, 55.0% at 18 months and 50.4% at the final 24-month assessment (Fig. S1). For the HC sample, 74.2% completed the 6-month follow-up assessment and 73.5% the 12-month assessment (Fig. S2). If the COVID-19 pandemic disrupted follow-up assessments, they were conducted remotely instead of in person.

### Socio-demographic characteristics

We aimed to recruit a HC sample that would be socio-demographically similar to the CHR sample, but the groups slightly differed in age (23.7 versus 22.4), years in education (15.9 versus 14.1 for CHR) and estimated IQ (112.6 versus 105.0). HC were also more likely than CHR participants to be in education (64.1% versus 48.7%) or/and employment (77.1% versus 49.7%), and to be in a relationship (43.0% versus 23.7%) (Table 1).

### Clinical characteristics

**Fig. 2:**
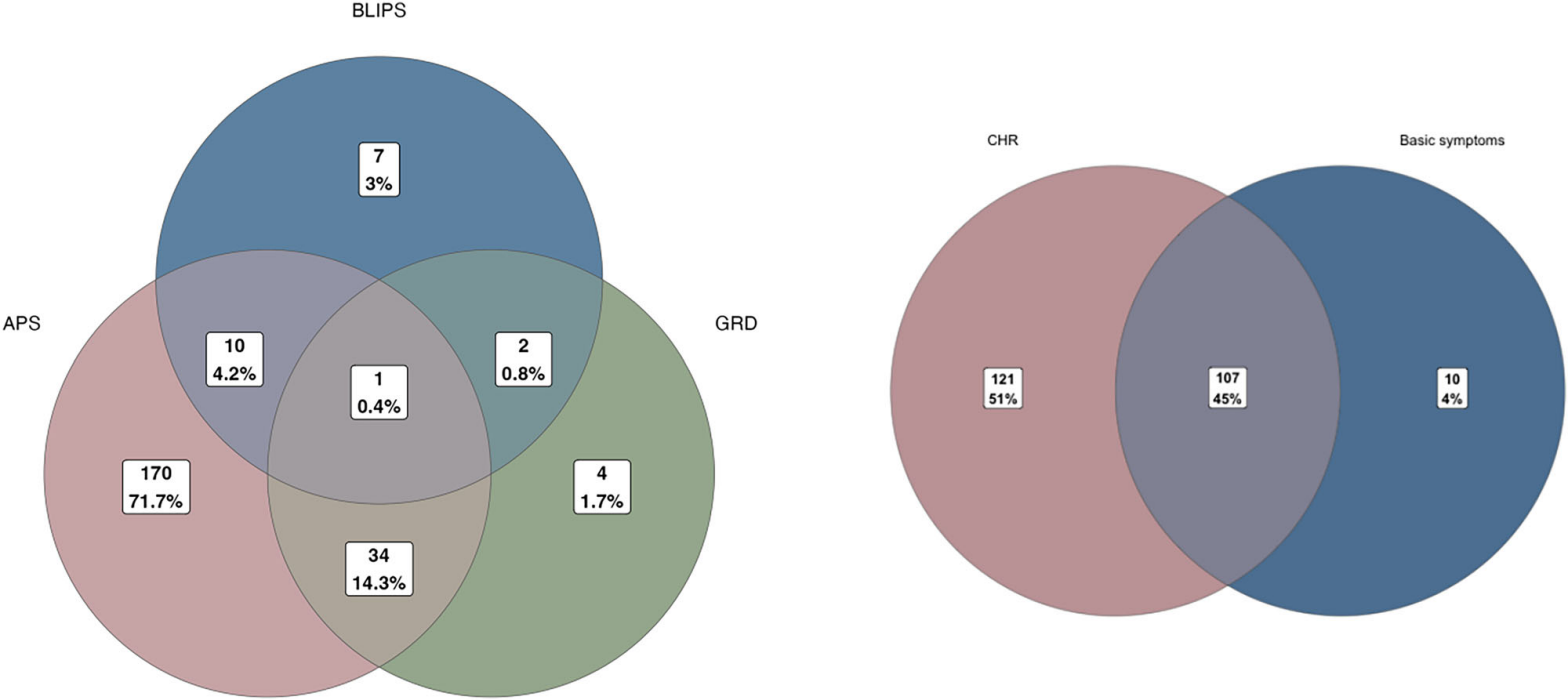
Distribution of CAARMS and SPI-A subgroups. Distribution of CAARMS and SPI-A subgroups. Left: Distribution of CAARMS subgroups: Attenuated Psychotic Symptoms, GDR, Genetic and Deterioration Syndrome, Brief Limited Intermittent Psychotic Symptoms. Right: Distribution of inclusion criteria.

In total, 228 CHR participants met CAARMS CHR criteria, 117 met Basic Symptoms criteria and 107 met both (Table 2, Fig. 2). Of the 228 participants that met the CAARMS inclusion criteria, 215 (94.3%) had attenuated psychotic symptoms, while 41 (18.0%) had trait liability (SPD or a first-degree relative with psychosis) and functional decline, and 20 (8.8%) had BLIPS. Most of the subjects with trait liability and BLIPS also had attenuated symptoms: only 11 of 228 participants who met the CAARMS criteria did so on the basis of genetic risk or BLIPS alone. Ten participants (4.2%) met Basic Symptoms criteria only.

**Table 2:** Clinical characteristics of individuals at clinical high-risk at baseline.

	*N* (%)
Family history of psychosis (1^st^ degree)	36 (15.1)
Present/past psychological intervention	162 (68.1)
Current psychotropic medication	
Antidepressants	92 (38.7)
Antipsychotics	33 (13.9)
Benzodiazepines	39 (16.4)
Mood stabilizers	8 (3.4)
Other psychotropics	2 (0.8)
Psychostimulants	3 (1.3)
Comorbidities	
Mood Disorders	126 (52.9)
Anxiety Disorders	127 (53.4)
Eating Disorders	20 (8.4)
Somatoform Disorders	11 (4.6)
Attempted suicide (lifetime)	59 (24.8)

The mean baseline SOFAS score for all participants was 53.7 (SD = 11.4). Mean baseline CAARMS severity scores (severity*frequency scores) for all participants across the four psychosis items were 11.4 (SD = 9.4) for unusual thought content, 13.4 (SD = 9.3) for non-bizarre ideas, 10.9 (SD = 7.1) for perceptual abnormalities, 6.8 (SD = 6.6) for disorganised speech, and 42.5 (SD = 20.3) for the total CAARMS positive.

Many CHR individuals also met DSM-IV criteria for an anxiety disorder (53.4%) or for a mood disorder (52.9%) (Table 2, Fig. S3), far fewer met criteria for an eating (8.4%) or a somatoform disorder (4.6%). Over a third of the CHR sample (38.7%) were taking antidepressant medications. A minority were taking antipsychotic medications (13.9%) or benzodiazepines (16.4%).

There were no significant differences in substance use between HC and CHR over the previous 3 months or the participant’s lifetime, except that HC individuals (89.9%) were more likely to have reported alcohol use in the last 3 months compared with CHR (73.7%). However, there was a higher proportion of moderate risk for tobacco abuse in CHR (46.0%) than HC (35.1%) (Table 3).

**Table 3:** HC and CHR lifetime and last 3 months substance use and abuse at baseline.

	HC, *N* (%)	CHR, *N* (%)	*p*-value^1^
***Use - Lifetime***			
Tobacco, *n* (%)	86 (64.2)	134 (58.0)	0.294
Alcohol, *n* (%)	120 (90.2)	197 (85.3)	0.233
Cannabis, *n* (%)	77 (57.5)	129 (55.8)	0.787
Other, *n* (%)	47 (35.3)	97 (42.0)	0.255
***Use - Last 3 months***			
Tobacco, *n* (%)	45 (37.8)	102 (49.5)	0.054
Alcohol, *n* (%)	116 (89.9)	165 (73.7)	**<0.001**
Cannabis, *n* (%)	32 (27.4)	75 (36.2)	0.131
Other, *n* (%)	36 (35.3)	83 (44.1)	0.181
***Moderate risk abuse***			
Tobacco, *n* (%)	47 (35.1%)	109 (46.0%)	**0.041**
Alcohol, *n* (%)	36 (28.8%)	77 (36.7%)	0.140
Cannabis, *n* (%)	68 (66.7%)	123 (64.1%)	0.700

*CHR* clinical high risk for psychosis; *HC* healthy controls. ^1^Pearson’s Chi-squared test.

Statistically significant comparisons are shown in bold.

The most frequent sources of referral were community mental health teams (47.2%), social services or supported accommodation (15.0%) and general practitioner (14.0%) (Supplementary Table S3).

### Follow-up rates and transition status

Among the 238 CHR participants recruited to the study, 202 (84.9%) participated in at least one follow-up assessment and EHRs (available from nine sites) were accessed for 209 (87.8%) participants. Using these two data sources, follow-up data were available for 224 (94.1%) of CHR individuals, followed for a mean of 691.6 (SD = 438.1) days.

**Fig. 3:**
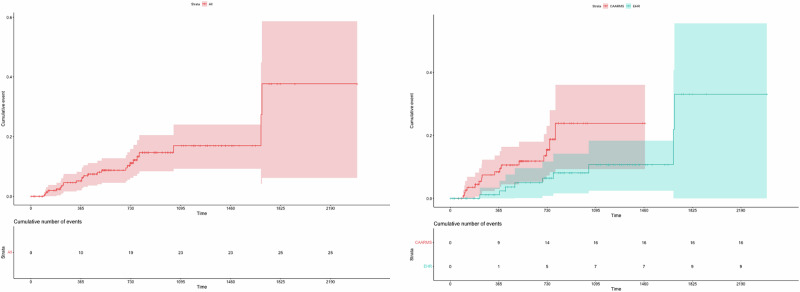
Kaplan-Meier survival curve and Greenwood 95% confidence intervals showing cumulative probability of developing a psychotic disorder in the entire CHR group (left) and stratified by source of follow-up: psychometric assessment with the CAARMS or electronic health records (EHR) (right).

In total, 25 (10.5%) CHR individuals developed a FEP; 16 of these transitions (64.0%) were defined psychometrically using the CAARMS and nine (36.0%) were defined clinically through clinical data in EHRs (see Table S4 for a descriptive comparison of demographic and clinical characteristics between those who transitioned and those who did not). The cumulative incidence of psychosis was 0.019 (95%CI: 0.000-0.038, 196 individuals still at risk) at 6 months, 0.051 (95%CI: 0.020–0.082, 169 individuals still at risk) at 12 months, 0.087 (95%CI: 0.045–0.127, 133 individuals still at risk) at 18 months and 0.111 (95%CI: 0.062–0.158, 102 individuals still at risk) at 24 months, 0.170 (95%CI: 0.092–0.240, 37 individuals still at risk) at 36 months, 0.170 (95%CI: 0.092–0.240, 15 individuals still at risk) at 48 months, 0.377 (95%CI: 0.062-0.587, 3 individuals still at risk) at 60 months, 0.377 (95%CI: 0.062–0.587, 2 individuals still at risk) at 72 months (Fig. 3).

EHR-based follow-up was associated with a lower risk of transition compared to those followed-up using the CAARMS (HR = 0.34, 95%CI: 0.13–0.87, *p* = 0.025; Fig. 3). There was a significantly lower proportion of female participants represented in EHR follow-up compared to those followed up using the CAARMS alone (*p* = 0.04) and differences in ethnicity (*p* < 0.001), largely driven by lower proportions of Asian participants and higher proportions of Black participants in EHR follow-up. There were no differences in age, baseline CAARMS or baseline SOFAS scores between the two groups (*p* > 0.05). The difference in transition risk between EHR- and CAARMS-based follow-up was no longer significant when adjusted for sex and ethnicity (adjusted hazard ratio, aHR=0.47, 95%CI: 0.17–1.34, *p* = 0.16).

### Symptomatic and functional outcomes at follow-up

Clinical assessments were available for 202 CHR participants who attended at least one follow-up assessment; the mean time between the baseline and the last observed clinical assessment for these participants was 546 (SD = 325.9) days. After excluding those who transitioned to psychosis (irrespective of data source), 78/180 (43.3%) CHR individuals continued to meet CHR status at their final clinical follow-up whilst 102/180 (56.7%) showed remission from the CHR state.

Of those who attended at least one follow-up assessment, 19/202 (9.4%) CHR individuals had clinically determined good functioning at baseline and 80/202 (39.6%) at their last follow-up visit. There was a significant increase in SOFAS scores from 53.6 to 64.4 (mean difference = 10.9, 95%CI: 9.0–12.9, *p* < 0.001). Similarly, there was a significant decrease in positive symptom severity across all CAARMS positive items (unusual thought content [mean difference = 7.1, 95%CI: 5.7–8.5, *p* < 0.001]; non-bizarre ideas [mean difference = 7.1, 95%CI: 5.6–8.6, *p* < 0.001]; perceptual abnormalities [mean difference = 5.8, 95%CI:4.7-7.0, *p* < 0.001]; disorganised speech [mean difference = 2.9, 95%CI:2.0–3.9, *p* < 0.001]) and total CAARMS positive (mean difference = 23.0, 95%CI: 19.4–26.5, *p* < 0.001).

## Discussion

A large sample of 238 CHR for psychosis and 134 HC were recruited and included into the PSYSCAN study. A comprehensive number of clinical measures were collected at baseline and 5 follow-up timepoints generating a rich and well-characterised dataset. The sample was characterised by a high prevalence of psychiatric comorbidities, with more than half of CHR participants presenting with a baseline diagnosis of an anxiety or mood disorder. The pattern of comorbidities was highly heterogeneous, also including post-traumatic stress disorder, eating disorders, and somatoform disorders. This aligns with cumulative evidence demonstrating that the CHR population is diverse in its clinical presentation^11^. It also supports the notion that psychosis onset may emerge from various non-psychotic precursors^46^, highlighting its inherently transdiagnostic nature. As a result, there have been recent calls to expand CHR assessments to include baseline evaluations of comorbid psychopathological dimensions^11^ and the development of early detection services for young people who are vulnerable to a range of adult psychiatric disorders, rather than just psychosis^47^. However, broadening the inclusion criteria for early detection teams can substantially increase the logistical demands on these clinical services, with much greater numbers of potential referrals^48^. The high prevalence of MDD among CHR individuals, particularly its greater prevalence in those who transitioned to psychosis compared to those who did not, is consistent with evidence suggesting that a history of depressive episodes may adversely affect the course of attenuated psychotic symptoms in CHR^49^. This aligns with the affective pathway to psychosis, which posits that affective dysregulation acts as the central link between early traumatic or stressful experiences and the onset of psychosis^14,50,51^.

There was a lack of significant differences in both recent and lifetime cannabis use between CHR subjects and HC. While this is in line with other studies^14^, it contrasts with the higher rates of cannabis use in people who have developed a psychotic disorder, which have consistently been found to be higher than in controls^52^. This difference in cannabis use between CHR and psychotic samples raises the possibility that in people with psychosis, cannabis use may partly be driven by effects of the disorder itself. Although cannabis use has been implicated as a risk factor for psychosis^53^, recent follow up studies in CHR samples have not found a significant association between cannabis use and later transition to psychosis^54–56^.

Over 90% of the CHR individuals were followed up either face to face or remotely. The rate of transition to psychosis was relatively low (10.5%), but similar to that in other recent multi-centre studies^57,58^. Previous meta-analytical work suggests that transition to psychosis in CHR samples may be declining, possibly due to improved clinical engagement, active interventions, and early detection^59^. This presents both a conceptual and analytical challenge, as the relatively low rate of transition limits the statistical power to identify predictors of psychotic disorder as an outcome. Potential approaches to address this issue include exploring long-term transition risk^15^, investigating transdiagnostic outcomes such as socio-occupational functioning^60^, or broadening the transdiagnostic inclusion criteria for psychosis-risk populations^61^.

In previous prospective studies of CHR samples and in a recent meta-analysis^23^, there was an overall improvement in both symptom severity and level of functioning subsequent to baseline. Among those who did not develop psychosis, a substantial proportion (43.3%) had not achieved symptomatic remission (defined as no longer meeting criteria for the CHR state) at the last available follow-up, or had not shown an improvement in their level of functioning (39.6%)^42^. Thus, despite a group-level improvement in clinical and functional status over time, a large proportion of our CHR sample that did not become psychotic had poor clinical and/or functional outcomes.

### Strengths and limitations

This study has several strengths. Firstly, we recruited a relatively large sample of well-characterised CHR and HC, retaining a large proportion of them into the study across multiple follow-ups. PSYSCAN participants were recruited across 12 sites located across four continents. CHR participants can be difficult to recruit to research studies. Multi-centre studies like PSYSCAN provide a way to enrol large CHR samples but are logistically demanding and require substantial funding. In the present study, retention rates for CHR were satisfactory, with up to 61% completing the 12-month follow-up assessment and 50% completing follow up at 24 months (or later). This may have been due to the higher frequency of follow-up assessments compared to previous large studies^62^ as well as the use of EHR to supplement missing data.

There are also several limitations. Firstly, some sites recruited a small number of participants, introducing the potential for confounding site effects^30^. Many centres do not have clinical early detection services for CHR individuals, and within the PSYSCAN consortium, sites that lacked this specialised infrastructure recruited significantly fewer participants. Even when such services are well-established, CHR individuals may be referred to other clinical teams^63^. In the present study, we sought to minimise these effects by standardising assessments and protocols, and regularly training study researchers in their use. Secondly, proportion who transitioned to psychosis was relatively small (10%), although in line with similar large studies^57,58^. While this makes the prediction of psychosis transition difficult to analyse, we do have a well characterised group of CHR and it will be possible to assess how accurately other important outcomes such as symptom remission and functioning can be predicted from baseline multimodal data. Nevertheless, the inability to assess individuals lost to follow-up may introduce potential bias in subsequent analyses to identify predictors of outcomes. Finally, despite the onset of COVID-19 pandemic during the follow up phase of the study, most sites were able to continue with clinical assessments by conducting these remotely instead of face-to-face. However, delays in obtaining approval to conduct the assessments remotely during this period likely extended assessments dates for some participants. Additionally, the stress and uncertainty associated with the pandemic could have exacerbated symptoms of psychosis and general psychopathology^64^, potentially impacting the clinical measures captured during this period.

## Conclusions

Consistent with other recent multi-centre studies, the PSYSCAN CHR cohort is characterised by high levels of psychiatric comorbidity and relatively low rates of transition to psychosis. The core aim of the PSYSCAN study is to integrate neuroimaging, clinical, cognitive, and peripheral biomarker data to facilitate the prediction of clinical and functional outcomes in CHR individuals. A large sample of individuals at CHR for psychosis and HC was recruited and assessed at multiple time points. The study has generated a multi-modal dataset that will be used to identify predictors of outcomes in this population.

## Supplementary information

Supplementary material revised

## Data Availability

The data that support the findings of this study are available from the corresponding author upon reasonable request.
