# Supplementary material for: PSYSCAN multi-centre study: baseline characteristics and clinical outcomes of the clinical high risk for psychosis sample
Source: Schizophrenia (Heidelb). 2025 Apr 17;11(1):66. doi: 10.1038/s41537-025-00598-x (PMC12006469; doi:10.1038/s41537-025-00598-x)
Supplement: Supplementary file 1 — Supplementary material revised [file 41537_2025_598_MOESM1_ESM.docx]

| **Table S1: Visits and study procedures for CHR** | | | | | | | |
| --- | --- | --- | --- | --- | --- | --- | --- |
|  |  |  |  |  |  |  |  |
| **VISIT NUMBER** | **1** | **2** | **3** | **4** | **5** | **6** |  |
| **Assessments** | **BL** | **3 m**  **(+/- 1m*)** | **6 m**  **(+/- 1m*)** | **12 m**  **(+/- 1m*)** | **18 m**  **(+/- 1m*)** | **24 m**  **(+/- 2m*)** |  |
| Informed consent | X |  |  |  |  |  |  |
| In- and exclusion criteria | X |  |  |  |  |  |  |
| Sociodemographics | X |  |  |  |  |  |  |
| Medical history | X |  |  |  |  |  |  |
| Physical health | X | X | X | X | X | X |  |
| Current medication use | X | X | X | X | X | X |  |
| Recent psychiatric history | X | X | X | X | X | X |  |
| **Clinical Assessments** |  |  |  |  |  |  |  |
| CHRA Part 1 | X | X | X | X | X | X |  |
| CHRA Part 2 | X |  | X | X |  | X |  |
| HAM-D | X |  | X | X |  | X |  |
| ASSIST 3.0 | X | X | X | X | X | X |  |
| PAS | X |  |  |  |  |  |  |
| CUQ | X |  |  |  |  |  |  |
| **Self-Report Measures** |  |  |  |  |  |  |  |
| CTQ | X |  |  |  |  |  |  |
| RSA | X |  |  |  |  |  |  |
| EHI | X |  |  |  |  |  |  |
| **Cognitive Measures** |  |  |  |  |  |  |  |
| PAL | X |  | X | X |  | X |  |
| SSP | X |  | X | X |  | X |  |
| RVP | X |  | X | X |  | X |  |
| ERT | X |  | X | X |  | X |  |
| WAIS-III (abbreviated) | X |  |  |  |  |  |  |
| HVLT-R | X |  | X | X |  | X |  |
| **Neuroimaging** |  |  |  |  |  |  |  |
| Structural MRI, resting-state fMRI, and diffusion tensor MRI | X |  | X | X |  |  |  |
| **Bloods and Biomarkers** |  |  |  |  |  |  |  |
| Plasma and serum extraction from blood to measure genetic, immune, proteomic, and metabolic markers | X |  | X | X |  |  |  |

* Timing is relative to the baseline visit.; m, month; BL, Baseline; CUQ, Cannabis Use Questionnaire; EHI, Edinburgh Handedness Inventory; CTQ, Childhood Trauma Questionnaire; RSA, Resilience Scale for Adults; PAS, Premorbid Adjustment Scale; CHR, Clinical High Risk; HAM-D, Hamilton Rating Scale for Depression; WHO, World Health Organisation; PANSS, Positive And Negative Syndrome Scale; CGI, Clinical Global Impression scale; YMRS, Young Mania Rating Scale; CANSAS-P, Camberwell Assessment of Need Short Appraisal Schedule; HVLT-R, Hopkins Verbal Learning Test-Revised; WAIS, Wechsler's Adult Intelligence Scale; MRI, Magnetic Resonance Imaging.

| Table S2: Visits and study procedures for HC. | | | |
| --- | --- | --- | --- |
| VISIT NUMBER | 1 | 2 | 3 |
| Assessments | **BL** | **6 months**  **+/- 1 month** | **12 months**  **+/- 1 month** |
| Informed consent | X |  |  |
| In- and exclusion criteria | X |  |  |
| Sociodemographics | X |  |  |
| Medical history | X |  |  |
| Physical health | X | X | X |
| Current medication use | X | X | X |
| Clinical Assessments |  |  |  |
| CHRA Part 1 | X |  | X |
| CHRA Part 2 | X |  | X |
| HAM-D | X |  |  |
| ASSIST 3.0 | X | X | X |
| PANSS | X |  | X |
| CGI | X |  |  |
| YMRS | X |  |  |
| CANSAS-P | X |  |  |
| Cannabis Use Questionnaire | X |  |  |
| PAS | X |  |  |
| Self-Report Measures |  |  |  |
| CTQ | X |  |  |
| RSA | X |  |  |
| EHI | X |  |  |
| Cognitive Measures |  |  |  |
| PAL | X | X | X |
| SSP | X | X | X |
| RVP | X | X | X |
| ERT | X | X | X |
| WAIS-III (abbreviated) | X |  |  |
| HVLT-R | X |  |  |
| Neuroimaging |  |  |  |
| Structural MRI, resting-state fMRI, and diffusion tensor MRI | X | X | X |
| Bloods and Biomarkers |  |  |  |
| Plasma and serum extraction from blood to measure genetic, immune, proteomic, and metabolic markers | X | X | X |

* Timing is relative to the baseline visit. BL, Baseline; HC, healthy control; EHI, Edinburgh Handedness Inventory; CTQ, Childhood Trauma Questionnaire; RSA, Resilience Scale for Adults; PAS, Premorbid Adjustment Scale; CHR, Clinical High Risk; HAM-D, Hamilton Rating Scale for Depression; WHO, World Health Organisation; PANSS, Positive And Negative Syndrome Scale; CGI, Clinical Global Impression scale; YMRS, Young Mania Rating Scale; CANSAS-P, Camberwell Assessment of Need Short Appraisal Schedule; HVLT-R, Hopkins Verbal Learning Test-Revised; WAIS, Wechsler's Adult Intelligence Scale; MRI, Magnetic Resonance Imaging.

**CHR and HC Inclusion/Exclusion Criteria:**

**Inclusion criteria**

Inclusion criteria for all subjects (clinical high risk individuals and healthy controls):

- 16-40 years old
- Written informed consent of subjects aged 18 to 40 years
- Written informed consent of parents and/or legal guardians for subjects aged 16 or 17, in addition to assent from the minor subject, following local laws and regulations

Participants at high risk of psychosis will be evaluated using a quantitative clinical tool that assesses:

- Inclusion into one of three groups as assessed by the Comprehensive Assessment of At-Risk Mental States (CAARMS version 2006)^1^: i) vulnerability group, ii) attenuated psychosis group, iii) brief intermittent psychosis symptoms group.
- Inclusion based on meeting criteria for “basic symptoms” which are assessed using the Schizophrenia Proneness Instrument, (SPI-A)^2^.

Inter-rater reliability was ensured throughout via an on-going training programme supported by PSYSCAN.

**Exclusion criteria**

Exclusion criteria for all subjects (patients and healthy controls):

- Any previous neurosurgery or neurological disorder, including epilepsy
- History of head injury resulting in unconsciousness lasting at least 1 hour
- Pregnancy
- Any contraindications for MRI
- Refusing to have blood drawn and/or MRI performed
- Subject is unable to fully comprehend the purpose of the study or make a rational decision whether or not to participate
- Estimate of IQ < 70
- Antipsychotic medication for > 30 days (cumulative number of days) in the 3 months before the baseline assessments (including self-ratings and screening assessments), at doses that would be adequate for treating a first episode of psychosis (i.e. excludes very low doses)
- Any past episode of frank psychosis lasting > 7 days

**
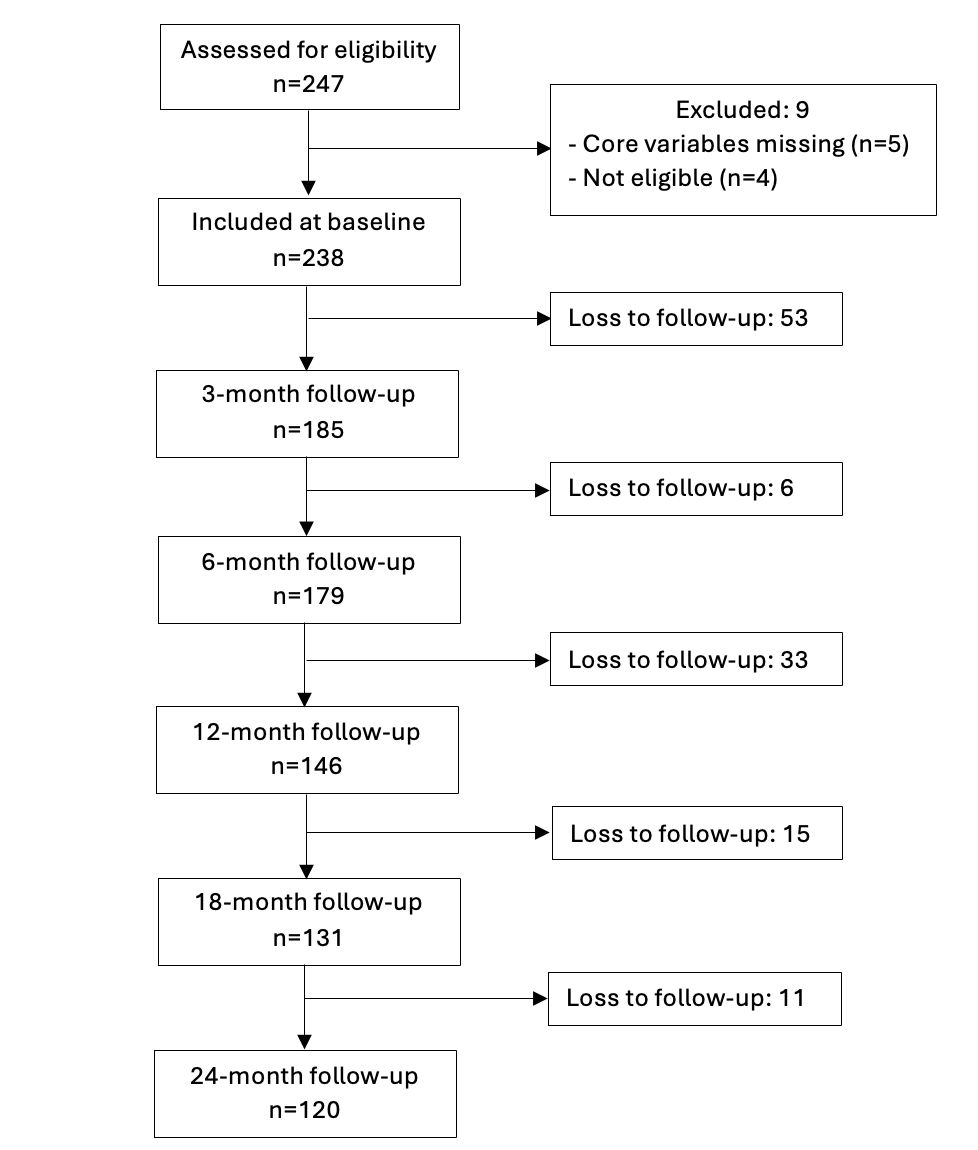
Figure S1. CONSORT flow diagram displaying the progress of CHR through the study.**

**Figure S2. CONSORT flow diagram displaying the progress of HC through the study.**

**
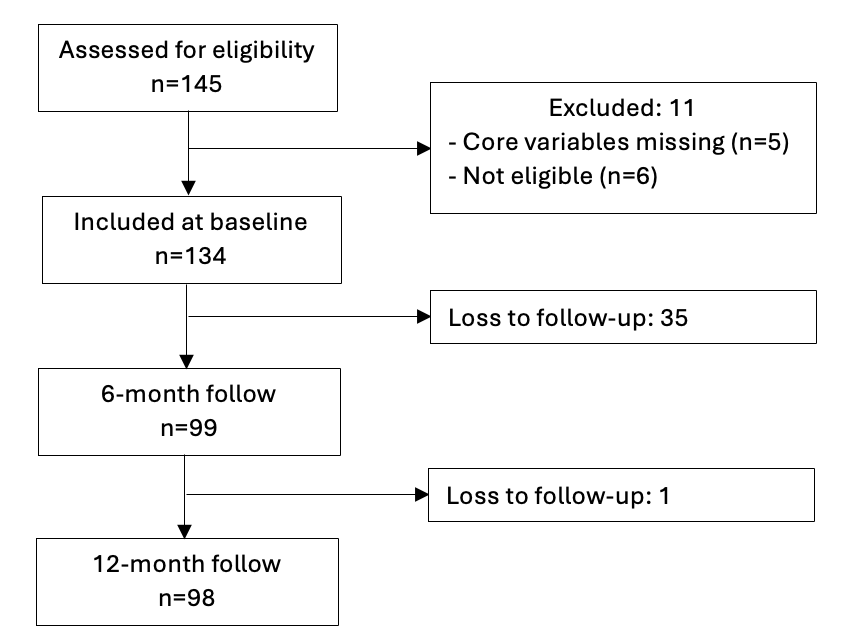
**


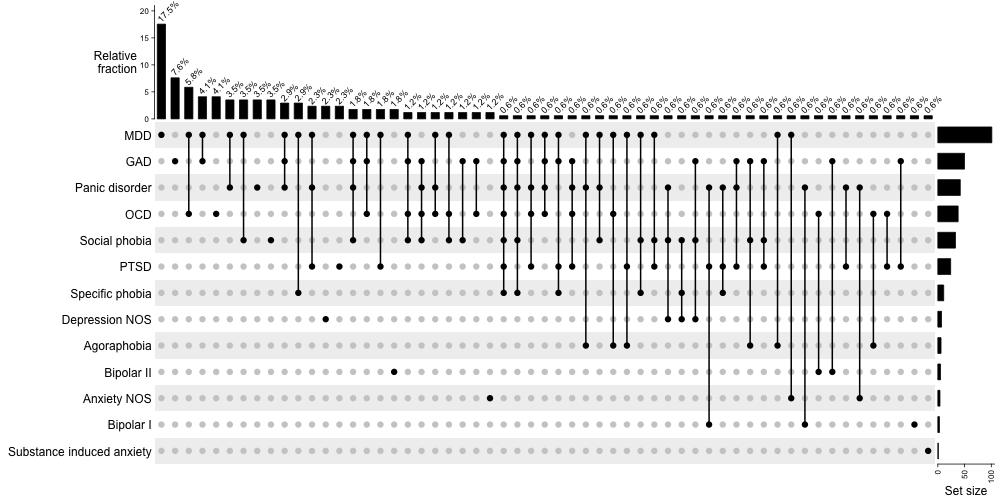
**Figure S3: Comorbid diagnoses distribution for CHR**

Vertical bars show the relative fraction (%) of each combination; horizontal bars show the total number of individuals with each diagnosis alone. Abbreviations: CHR, clinical high risk; MDD, major depressive disorder; GAD, generalised anxiety disorder; OCD, obsessive compulsive disorder; PTSD, post-traumatic stress disorder; NOS, not otherwise specified.

**Table S3: Source of referral.**

| **Source of referral** | **N (%)** |
| --- | --- |
| A&E | 4 (1.9%) |
| CAMHS | 18 (8.4%) |
| Carers or relatives | 5 (2.3%) |
| CMHT | 101 (47.2%) |
| EI | 5 (2.3%) |
| GP | 30 (14.0%) |
| Inpatient Mental Health Services | 2 (0.9%) |
| Other | 4 (1.9%) |
| Physical health | 1 (0.5%) |
| Schools or colleges | 10 (4.7%) |
| Self | 2 (0.9%) |
| Social services or supported accommodation | 32 (15.0%) |
| Unknown | 23 |

A&E: Accident and Emergency; CAMHS: Child and Adolescent Mental Health Services; CMHT: Community Mental Health Team; EI: Early Intervention; GP: General Practitioner. Categories generated based on ^3^

**Table S4.** **Sociodemographic and clinical characteristics of individuals at clinical high-risk who transitioned and did not transition to psychosis.**

|  |  | CHR-NT  (N=213) | CHR-T  (N=25) |
| --- | --- | --- | --- |
| Age, mean (SD) |  | 22.0 (4.8) | 22.0 (4.0) |
| Sex, n (%) | Female | 101 (52.4) | 9 (36.0) |
|  | Male | 111 (47.6) | 16 (64.0) |
| Ethnicity, n (%) | Asian Indian | 8 (3.8) | 0 (0) |
|  | Black | 23 (10.8) | 1 (4.0) |
|  | East Asian | 31 (14.6) | 8 (32.0) |
|  | White | 129 (60.8) | 13 (52.0) |
|  | Other | 21 (9.9) | 3 (12.0) |
|  | Missing | 1 (0.5) | 0 (0) |
| Relationship status, n (%) | In a relationship | 50 (24.8) | 4 (16.0) |
|  | Single/divorced/separated | 150 (74.3) | 21 (84.0) |
|  | Other | 2 (1.0) | 0 (0) |
|  | Missing | 10 (4.7) | 0 (0) |
| Current living conditions, n (%) | Living alone | 23 (11.1) | 2 (8.0) |
|  | Living with family/partner/friends/other | 176 (85.0) | 22 (88.0) |
|  | Other | 8 (3.9) | 1 (4.0) |
|  | Missing | 5 (2.4) | 0 (0) |
| Education (years), mean (SD) | | 14 (3.3) | 13 (2.8) |
| Student, n (%) | No | 107 (51.4) | 12 (48.0) |
|  | Yes, full time | 82 (39.4) | 10 (40.0) |
|  | Yes, part time | 19 (9.1) | 3 (12.0) |
|  | Missing | 4 (1.9) | 0 (0) |
| Current employment, n (%) | Employed full-time | 58 (32.4) | 9 (42.9) |
|  | Employed part-time | 30 (16.8) | 2 (9.5) |
|  | Unemployed | 91 (9.1) | 10 (47.6) |
|  | Missing | 33 (15.6) | 4 (16.0) |
| Father - years of education, mean (SD) | | 14.8 (4.1) | 14.3 (4.0) |
| Mother - years of education, mean (SD) | | 15.0 (3.8) | 14.2 (4.2) |
| IQ, mean (SD) | | 105.0 (18.3) | 102 (13.8) |
| Current psychotropic medication | |  |  |
| Antidepressants | | 84 (56.8) | 7 (38.9) |
| Antipsychotics | | 26 (17.6) | 7 (38.9) |
| Benzodiazepines | | 34 (23.0) | 5 (27.8) |
| Mood stabilizers | | 7 (4.7) | 0 (0.0) |
| Other psychotropics | | 0 (0) | 0 (0) |
| Psychostimulants | | 2 (1.4) | 0 (0) |
| Comorbidities | |  |  |
| Mood Disorders | | 74 (39.2) | 14 (58.3) |
| Anxiety Disorders | | 88 (44.0) | 11 (44.0) |
| CAARMS, mean (SD) | |  |  |
| Total positive | | 46 (20.4) | 53 (22.2) |
| Unusual thought content | | 13 (8.8) | 15 (8.5) |
| Non-bizarre ideas | | 15 (8.4) | 15 (8.0) |
| Perceptual abnormalities | | 12 (6.19) | 16 (7.2) |
| Disorganised speech | | 8 (6.2) | 11 (7.3) |
| SOFAS, mean (SD) | | 72 (130.3) | 55 (11.7) |

**References**

1 Yung AR, Yuen HP, McGorry PD. Mapping the onset of psychosis: the Comprehensive Assessment of At-Risk Mental States. *Aust N Z J Psychiatry Nov-Dec* 2005; **39**: 964–971.

2 Schultze-Lutter F, Theodoridou A. The concept of basic symptoms: its scientific and clinical relevance. *World Psychiatry Feb* 2017; **16**: 104–105.

3 Fusar-Poli P, Rutigliano G, Stahl D, Schmidt A, Ramella-Cravaro V, Hitesh S *et al.* Deconstructing Pretest Risk Enrichment to Optimize Prediction of Psychosis in Individuals at Clinical High Risk. *JAMA Psychiatry* 2016; **73**: 1260–1267.
